# A Novel Decellularized Fibrocartilage Graft Promotes Tympanic Membrane Repair

**DOI:** 10.1002/adhm.202501774

**Published:** 2026-05-02

**Authors:** Paul M. Gehret, Ryan M. Friedman, Gautham Nair, Stephanie L. Fung, Dana D. Ragbirsingh, John Germiller, Riccardo Gottardi

**Affiliations:** ^1^ Department of Surgery Division of Otolaryngology Children's Hospital of Philadelphia Philadelphia Pennsylvania USA; ^2^ Department of Bioengineering School of Engineering and Applied Science University of Pennsylvania Philadelphia Pennsylvania USA; ^3^ Department of Otorhinolaryngology‐Head & Neck Surgery Perelman School of Medicine at the University of Pennsylvania Philadelphia Pennsylvania USA; ^4^ Department of Pediatrics Division of Pulmonary Medicine Perelman School of Medicine at the University of Pennsylvania Philadelphia Pennsylvania USA; ^5^ Ri.MED Foundation Palermo PA Italy

**Keywords:** cartilage, decellularized ECM, meniscus, otolaryngology, tympanic membrane

## Abstract

Tympanoplasty repairs tympanic membrane (TM) perforations using grafts. In pediatric patients, cartilage grafts are preferred over fascia due to superior mechanical properties that prevent complications from negative middle ear pressure, including graft failure, retraction, and cholesteatoma formation. However, autologous grafts cause donor‐site morbidity and increased surgical time. To overcome these limitations, we engineered an allogeneic porcine **men** iscus **d** ecellularized (MEND) fibrocartilage graft with a unique microchannel structure created by selective elastin and vascular digestion to promote host cell invasion and integration. We evaluated MEND in a rat TM acute perforation model, comparing outcomes to autologous cartilage and fascia grafts, the current clinical standards of care. TMs were monitored via otoendoscopy through 4 weeks, then analyzed by histology and immunohistochemistry. MEND and auricular cartilage grafts successfully closed perforations by day 3, outperforming fascia grafts which frequently dislodged due to poor mechanical integrity. However, unlike cartilage grafts, MEND fully remodeled by day 28, providing superior graft closure and tissue integration compared to both traditional materials. These findings demonstrate MEND's potential as an off‐the‐shelf solution for pediatric tympanoplasty.

## Graphical abstract

An off‐the‐shelf decellularized porcine meniscus fibrocartilage graft (MEND) is engineered for pediatric tympanoplasty. Featuring a microchannel architecture that promotes host cell invasion, MEND rapidly closes tympanic membrane perforations and fully remodels in vivo, outperforming fascia and matching cartilage grafts while avoiding donor‐site morbidity.

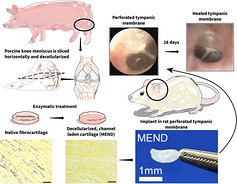

## 1 Introduction

Persistent tympanic membrane (TM) perforations occur in nearly 100 000 children each year [1, 2, 3, 4], and can lead to infections with otorrhea, pain with water ingress, and additional long‐term complications such as conductive hearing loss and cholesteatoma. Perforations cause hearing loss as a direct result of the reduced surface area and altered acoustics of the TM, and sometimes from tympanosclerosis, a chronic scarring process that can stiffen of the TM and ossicular chain [5, 6]. Cholesteatoma occurs when skin epithelium expands inwardly within the middle ear, either from direct epithelial invasion across a perforation defect, or from a retraction pocket forming in an intact TM [7]. Repair of TM perforation involves tympanoplasty or myringoplasty, surgical procedures in which a graft is inserted into the defect after first treating the edges of the perforation to promote a healing response. In tympanoplasty, the affected region of the TM is elevated and the middle ear exposed, in order to place the repair graft, typically medial to the TM; myringoplasty refers to repair through the perforation, without elevation of the TM itself. Most commonly, grafts are made from autologous cartilage or fascia harvested from nearby tissues [4, 8]. Cartilage is typically harvested from the tragus or the auricle (concha cymba or concha cavum [9]. Perichondrium from these same cartilages can also be used independently as a fascia graft. Fascia can also be harvested from the surface of nearby muscle, most commonly the temporalis major [10]. Pediatric tympanoplasty has failure rates of up to 20%, nearly 2.5‐fold worse than adult tympanoplasty [1, 4]. The primary cause of this disparity is believed to be Eustachian tube dysfunction, which is far more common in children. Eustachian tube dysfunction causes significant negative pressure in the middle ear that can dislodge a graft into the middle ear, resulting in surgical failure [4, 11, 12, 13, 14], and in severe cases can lead to acquired cholesteatoma from the formation of retraction pockets.

To mitigate risks of surgical failure due to this negative pressure, cartilage grafts are often preferred over fascia for pediatric TM repair because their higher biomechanical stiffness resists the negative pressure associated with Eustachian tube dysfunction [9, 15, 16, 17, 18]. However, there are disadvantages to autologous cartilage. Cartilage in the TM typically does not resorb and fails to remodel and recapitulate the native TM extracellular matrix (ECM) structure [9]. While this might be advantageous in the short term, nearly all children eventually outgrow Eustachian tube dysfunction, and thus do not need their TM to remain cartilaginous long term. Moreover, in some cases TMs fail to heal around the edges of the cartilage graft, or delayed secondary perforations can occur at the interface between native TM tissue and the cartilage matrix [18]. Revision surgery in such cases is made much more difficult by the stiffness and thickness of the persisting cartilage. Finally, the middle ear is more difficult to monitor for disease in a permanently cartilaginous TM, due to its thickness and opacity, and the fact that tympanometry is unreliable due to the stiffness of the cartilage [9, 18]. By contrast, fascia grafts, while mechanically inferior, are advantageous due to their fibrous ECM structure, which resembles the native TM and allows for scaffold integration and remodeling.

Given all these considerations, an ideal pediatric tympanoplasty graft would possess both the initial mechanical strength of cartilage, required to resist the negative pressure and remain adherent to the TM, and a fibrous ECM structure like fascia, capable of rapid integration and eventual remodeling, thus becoming less stiff and opaque over time. Additionally, since both cartilage and fascia autografts require an additional surgical procedure for tissue harvesting, which increases donor site morbidity, surgical time, and healthcare costs, an ideal graft would be available as an off‐the‐shelf product.

Biomaterials such as alginate, hyaluronic acid, chitosan, silk, cellulose and collagen have been proposed as alternatives to autologous grafts [19, 20, 21, 22, 23, 24, 25, 26]; however, these materials lack the required mechanical strength, are difficult to handle during surgery, and show limited integration. One class of materials that has become attractive as a tissue engineering scaffold is decellularized tissue‐derived ECM, which have proven successful, safe, and cost‐effective in various clinical applications [27, 28]. While the exact mechanism by which decellularized ECM grafts promote tissue repair and constructive remodeling is not fully understood, advantages of decellularized tissues include their high rate of graft acceptance, retention of native tissue composition and structure, and ability to support host cell attachment and infiltration [27]. Since cartilage autografts are commonly used for tympanic membrane repair, we engineered a xenogeneic decellularized graft using porcine meniscal fibrocartilage for pediatric TM repair. Our decellularized porcine meniscal fibrocartilage graft, termed MEND (MENiscus Decellularized), has a tensile strength comparable to that of autologous cartilage grafts currently used clinically, while also possessing a fibrous ECM structure more similar to the native TM [29]. These properties confer to MEND the potential to withstand the middle ear pressure which typically causes fascia grafts to fail in children, while simultaneously still allowing for good graft integration and remodeling.

In this work, MEND was fabricated via selective enzymatic removal of blood vessels and elastin fibers that are uniquely present in the fibroelastic cartilage of the meniscus [30]. After selective elastin digestion, the collagen fibers, proteoglycans, and glycosaminoglycans that are left in the construct are sieved with an abundance of microchannels ready for recellularization. First, we compared MEND histologically, biochemically, and mechanically to the gold standard grafts for tympanoplasty: auricular cartilage and fascia. Then, we leveraged a rat model of acute TM perforations to assess tissue repair and graft integration of MEND, auricular cartilage, and fascia over 4 weeks. We demonstrate that MEND promotes TM healing at a rate faster than fascia and similar to cartilage, but with increased graft integration and remodeling. Moreover, MEND maintained its initial mechanical properties after in vivo implantation, a property that could make it ideal for future use in the challenging environment of the pediatric middle ear.

## 2 Experimental Section

### 2.1 Decellularized Meniscus Synthesis

Menisci were obtained from a local abattoir scientific division (Animal Biotech Industries; Doylestown, PA) and frozen upon arrival at −80°C until use. Menisci were partially thawed at room temperature for 10 min then sliced sagittal in half, leaving 2 half‐crescent shaped pieces. The outermost horns were removed from each half and each piece was shaped into a rectangle. The tissue was mounted using superglue onto a vibratome (Lecia 1000S) and coronal sections of the meniscus were cut at 300 microns starting at the thickest end. Each rectangular section underwent 4 freeze thaw cycles with the final 2 cycles being conducted in a hypotonic buffer of 10 mM Tris‐base, pH 8. Next, the tissues were placed in an Erlenmeyer flask containing 10 mL of 0.1% (w/v) pepsin in 0.5 M acetic acid. The flask was shaken at 100 RPM for 24 h at 37°C. Following pepsin treatment, the tissues were washed 3 times with PBS for 24 h total. Elastase (0.03 U/mL) in 0.2 M Tris‐base, pH 8 was added to the flask and the tissues were shaken for 24 h. Tissues were again washed 3 times with PBS for 24 h total. The freshly decellularized meniscal sheets were stored at −20°C until use.

### 2.2 Histology of MEND

To prepare for histology, samples were fixed in 10% formalin then dehydrated in graded ethanol solutions with PBS. Three xylene (at room temperature) and paraffin washes (at 60°C) were conducted before embedding within paraffin. A microtome (Expredia HM 355S) was used to make 5‐micron tissue sections. Each section was stained with Hematoxylin & Eosin (H&E). After each stain, slides were dehydrated with ethanol and cleared in histoclear before mounting with limonene.

### 2.3 Thickness, Channel Diameter, and Porosity Measurements of MEND

H&E stained sections of the decellularized meniscal tissue were used to quantify thickness, channel diameter, and porosity. Each image was loaded on ImageJ where the linear measurement tool was utilized to measure the thickness of each tissue at 3 separate regions on the section. Additionally, this same tool was utilized to measure 50 random channels within each section. Finally, the total porosity was calculated by applying a threshold to each H&E image and the total “white space” was measured then divided by the overall area. This process was repeated on 3 sections per donor with a total of 5 donors.

### 2.4 DNA Quantification

Decellularized meniscus tissue was freeze dried with a lyophilizer, weighed, and digested at 10 mg/mL in 125 µg/mL papain with 5 mM EDTA and 5 mM L‐cysteine at 60°C. A PicoGreen assay (Thermofisher) was conducted by adding the diluted PicoGreen dye to 100 µL of the digestate and compared to the provided standard curve of λ‐DNA.

### 2.5 Tensile Testing

An Instron 5542 Material Tester was utilized to test 6 mm x 10 mm dog bone shaped native meniscus, decellularized meniscus, fascia, or ear cartilage. Prior to testing, samples were measured with a caliper then mounted on the Instron using a custom grip equipped with sandpaper. The sample was then preloaded to 0.15 N and tested until 10% strain at a rate of 0.5%/min. The slope of the most linear region of the stress–strain curve was employed to calculate the tensile modulus.

### 2.6 Rat Tympanoplasty Procedure

Twenty‐eight adult Sprague‐Dawley rats were used for this study (IAC 21–001431). Each rat was placed in an induction chamber with isoflurane then transferred to a nose cone to maintain anesthesia. Each ear was visualized with a 3 mm endoscope (Karl Storz) and a 22‐gauge needle was used to create an acute perforation in the posterior quadrant (approximately 20% of TM area). Then a graft of either MEND, ear cartilage, fascia, or no graft was placed on the defect. Grafts were the same diameter or slightly larger than the perforation. For MEND, a 1.5 mm biopsy punch was used to punch a small cylinder from the tissue sheet, perpendicular to the orientation of the microchannels whose orientation was in the plane of the sheet. For cartilage and fascia, a 1.5 mm biopsy punch was used to excise a small disc of full‐thickness auricular cartilage near the base of the ear, then the perichondrium and cartilage were separated under a stereomicroscope. The perichondrium was used as a fascia graft, and the remaining disc used as a cartilage graft. Each graft was placed on top of the defect then secured using several 1 mm^3^ cubes of gelfoam (Pfizer) moistened in 0.3% ofloxacin (Rising Pharmaceuticals, Inc). Control ears had no graft placed, but had the canal packed with gelfoam in the same manner. Rats were monitored via otoendoscopy until sacrifice at 4 weeks, then evaluated via histology and immunochemistry. Both ears were utilized as independent conditions in this study.

### 2.7 Histology and Immunostaining of Rat Tympanic Membranes

At each end point, the temporal bone of each the rat was dissected and fixed overnight in 10% formalin, then placed in a tube with 50 mL of formical‐2000 (Stat Labs) and shaken at 4°C for 2 weeks. Samples were then processed and stained as described above. Immunostaining was additionally conducted on the cross‐sections of each rat ear. Briefly, each section was deparaffinized then rehydrated into water. Antigen retrieval was conducted with Proteinase K in PBS (Qiagen) for 30 mins at room temperature then blocked using 5% bovine serum albumin (Fisher) in 0.1% Triton X100 (Sigma) in PBS for 2 h. Following blocking, the specific primary antibody diluted in blocking buffer was applied overnight at 4°C. Samples were washed 3x with PBS then the corresponding secondary antibody was applied diluted in blocking buffer for 2 h at room temperature. Finally, samples were washed 3x with PBS, counter stained with 300 nM DAPI in PBS, and mounted using fluoromount‐G (Invitrogen). All slides were imaged using a Keyence BX100 fluorescence microscope.

### 2.8 Ethical Statement

All animal experiments were conducted in accordance with the National Institutes of Health Guide for the Care and Use of Laboratory Animals and were approved by the Institutional Animal Care and Use Committee (IACUC) of Children's Hospital of Philadelphia (IAC 21–001431).

### 2.9 Statistical Analysis

Statistical analysis was performed and data were plotted in Prism GraphPad 9.0. All data were graphed and reported as means ± standard deviation. Total DNA content and porosity were analyzed using students’ t‐test, while one‐way analysis of variance (ANOVA) with Tukey's multiple comparisons was used to determine statistically significant differences for thickness and tensile strength. P < 0.05 is considered significant.

## 3 Results

### 3.1 Engineering a Channel‐Laden Decellularized Cartilage Graft for Tympanoplasty

**FIGURE 1:**
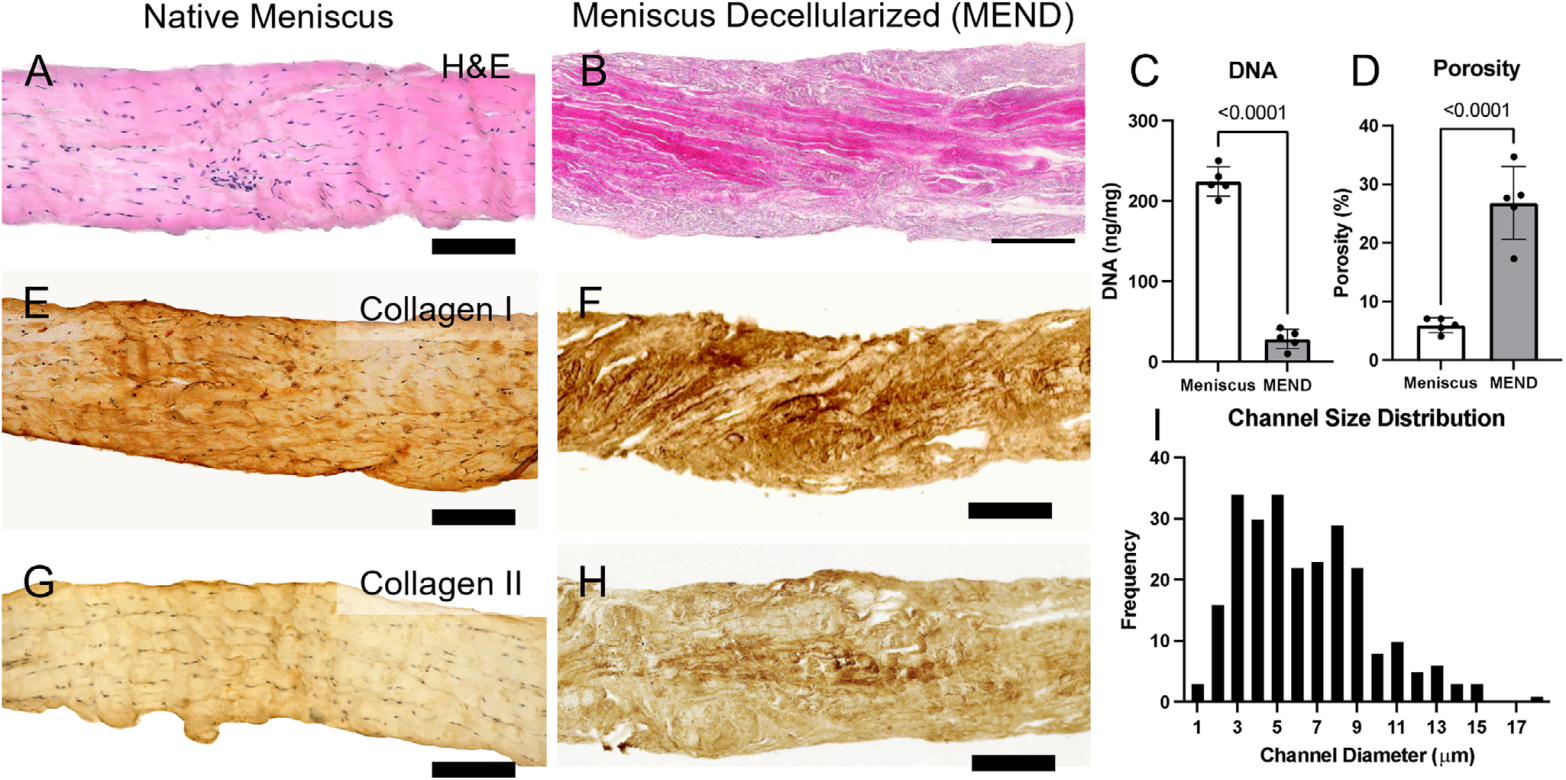
(A,B) H&E stained cross‐sections of the outer meniscus before and after digestion show removal of cell nuclei and significant channel creation. (C) Biochemical quantification of DNA content shows that MEND contains less than 50 ng DNA/mg tissue, which is the threshold for tissue decellularization. (D) Enzymatic‐based decellularization and removal of both blood vessels and elastin fibers leads to channel formation and an overall increase in tissue porosity. (E–H) Immunohistochemistry of collagen type I and collagen type II indicate that collagen content is not significantly altered during MEND fabrication. (I) MEND contains an abundance of channels throughout the matrix with an average diameter of 7.3 µm. Scale bars = 100 µm. Statistical analysis: students’ t‐test.

A pepsin and elastase mediated selective digestion was employed to decellularized the vibratome‐cut meniscus tissue as well as to create channels in the tissue. H&E‐stained sections from before and after pepsin‐ and elastase‐mediated decellularization show complete removal of existing cell nuclei (Figure 1A,B). Our methodology lead to near elimination of meniscal DNA, bringing it below the established threshold for tissue decellularization of 50 ng/mg (Figure 1C) [31]. Histology also indicates that the enzyme‐mediated digestion successfully removes elastin throughout the scaffolds (Figure S1), such that MEND contains an abundance of microchannels throughout the tissue (Figure 1A,B). As a result, tissue porosity increases from 7% in native tissue to 27% in MEND (Figure 1D). Collagen I and II content is conserved following our enzymatic decellularization method, but slight reorganization in the matrix structure is observed due to the creation of the channels (Figure 1E–H). These channels had an average diameter of 7.3 µm (Figure 1I) with an orientation predominantly in the plane of the MEND sheets (Figure 1B).

### 3.2 MEND Has a Fibrous Structure Similar to That of Native TM and Mechanical Strength Appropriate for Tympanoplasty

**FIGURE 2:**
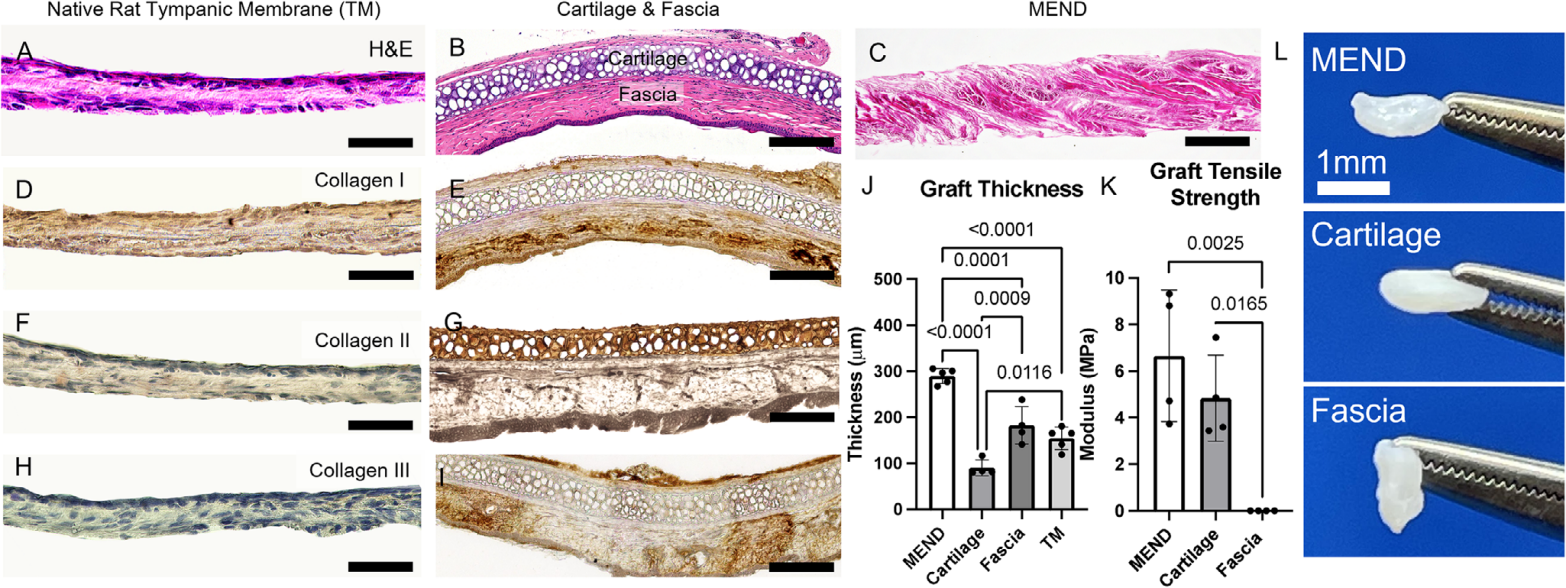
(A–C) H&E stained cross‐sections of the native rat tympanic membrane, ear cartilage and fascia, or MEND scaffolds show that both fascia and MEND are similar to the fibrous layer of the native tympanic membrane while cartilage has a dissimilar matrix structure. (D,E) Collagen type I, (F,G) collagen type II, and (H,I) collagen type III immunostaining shows that matrix components of auricular cartilage are significantly different from the native tympanic membrane. However, collagen type I content in the native rat tympanic membrane is like fascia. (J) Comparative graft thickness before to in vivo implantation shows that MEND is thicker than current grafting options in rat but is similar to the human tympanic membrane in terms of thickness. (K) Tensile modulus of each graft shows that cartilage and MEND have similar tensile strengths. (L) Macroscopic images of each graft held with ear forceps at the edge demonstrate that both MEND and cartilage grafts, but not fascia grafts, have enough rigidity to make them easy to handle. Scale bars = 100 µm for panel A‐I and 1 mm for panel L. Statistical analysis: ANOVA with Tukey's *post hoc*.

We assessed the ECM structure and biomechanical properties of MEND compared to the two gold standard TM grafts, auricular cartilage and fascia, via histology, immunostaining, and tensile testing, and related our findings to the native rat TM. H&E‐stained sections demonstrate that the rat TM is comprised of an epithelial, fibrous, and mucosal layer (Figure 2A). Healthy auricular cartilage and fascia (auricular perichondrium) tissues were sectioned together to easily visualize the strong differences in matrix composition and structure between these two tissues. Histology evidences that fascia has a fibrous ECM structure like the native TM, unlike cartilage, which contains cells surrounded by a dense cartilaginous matrix of collagen and glycosaminoglycans (Figure 2B). By comparison, MEND shares a similar fibrous structure with both the native TM and fascia (Figure 2C). Immunostaining shows that the tympanic membrane is strongly stained for collagen type I and II, with low signal for collagen III (Figure 2D,F,H). Fascia has a similar fibrous structure to the fibrous layer of the TM and also contains collagen type I, II, and III (Figure 2E,I). When comparing the major matrix collagens via immunohistochemistry, auricular cartilage primarily contained collagen type II (Figure 2G) with little collagen type I (Figure 2E) and type III (Figure 2I).

While MEND was thicker than autologous grafts measured from rats (Figure 2J), MEND thickness is closer to that of the human TM, which is also approximately 300 microns [32]. Finally, the tensile modulus of MEND was similar to that of auricular cartilage and significantly higher than fascia (Figure 2K), suggesting that MEND would be able to withstand negative ear pressure like cartilage grafts. Furthermore, MEND presented with more overall mechanical stability compared to fascia, as evidenced by resistance to bending under its own weight (Figure 2L). These biomechanical data further emphasize MEND's superior handleability for use in the clinic.

### 3.3 MEND Graft Implantation Promotes Wound Healing in a Rat Model of TM Perforation

**FIGURE 3:**
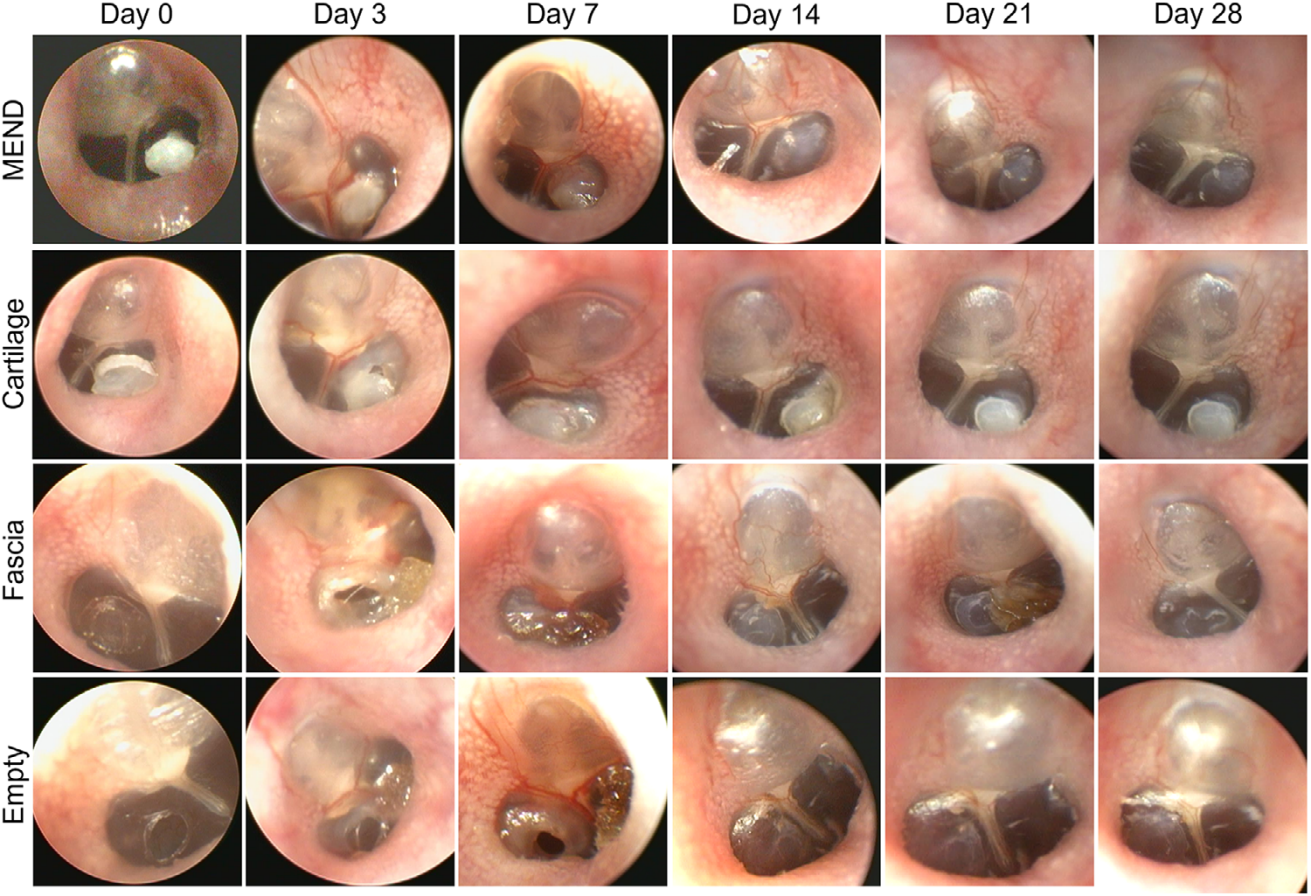
Periodic ear endoscopy following tympanoplasty with MEND, Ear cartilage, Fascia, and no graft (empty) shows rapid wound healing for MEND and cartilage‐treated ears but not for animals receiving fascia grafts or no treatment. MEND was readily integrated into the rat TM and continued to be remodeled until animal euthanasia. Ears were imaged at Day 0, 3, 7, 14, 21, and 28.

MEND, cartilage, or fascia grafts were implanted into a perforated rat TM. Following graft implantation, periodic endoscopic images were taken to monitor the integration and remodeling inside each rat ear. Figure 3 depicts one representative rat ear from each condition. By day 3, each graft was observed to be adherent and to cover the TM perforation; however, many of the fascia grafts were displaced from their original position. This is likely due to the poor mechanical properties of fascia, which makes graft placement challenging. In addition, all fascia grafts took at least one week to fully close the TM perforation. By contrast, both MEND and auricular cartilage closed the rat ear perforations within 3 days. Over the month‐long time course, auricular cartilage did not show significant remodeling and only minimal integration into the TM. This commonly occurs in pediatric patients with auricular cartilage grafts persisting as a “cartilage island” for the patient's entire life. However, MEND grafts were readily integrated into the TM and were continuously remodeled throughout the experimental timeline. This was evidenced by their gradual loss of opacity, such that the graft cannot be distinguished from native TM by week 3–4 (Figure 3 top row).

**FIGURE 4:**
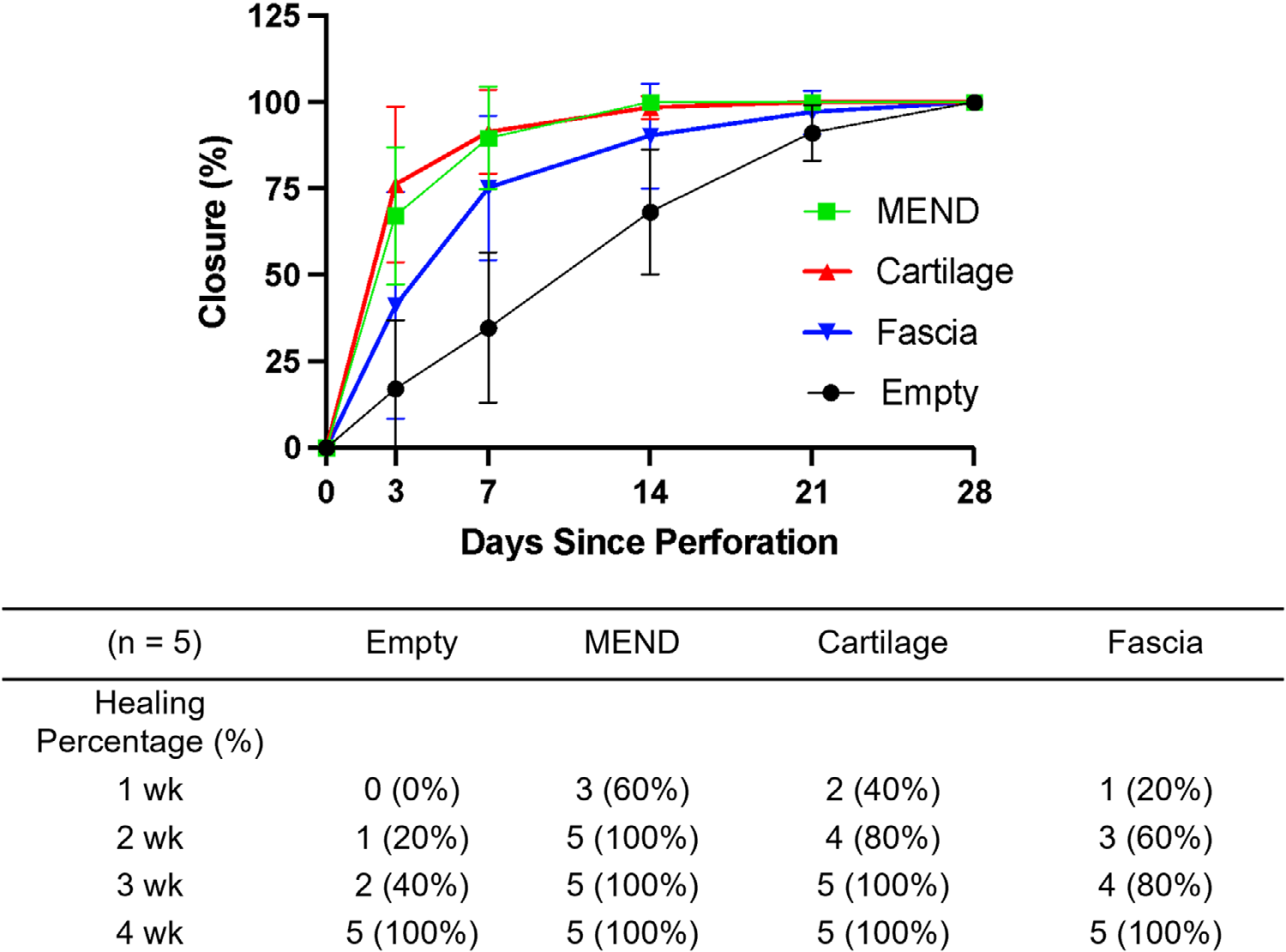
The percentage of rats with healed tympanic membrane perforation over time.

Untreated animals demonstrated TM wound closure within 2 to 3 weeks post‐perforation, indicating that healing with all 3 grafts was faster than the natural healing rate. MEND and cartilage had the fastest healing, with nearly 75% of all perforations closed by day 3 and 100% closed within 2 weeks. Healing was slower in the fascia group, which only reached 100% closure at 3 weeks (Figure 4).

### 3.4 MEND Repaired the Fibrous Structure of the TM Superior to Cartilage

**FIGURE 5:**
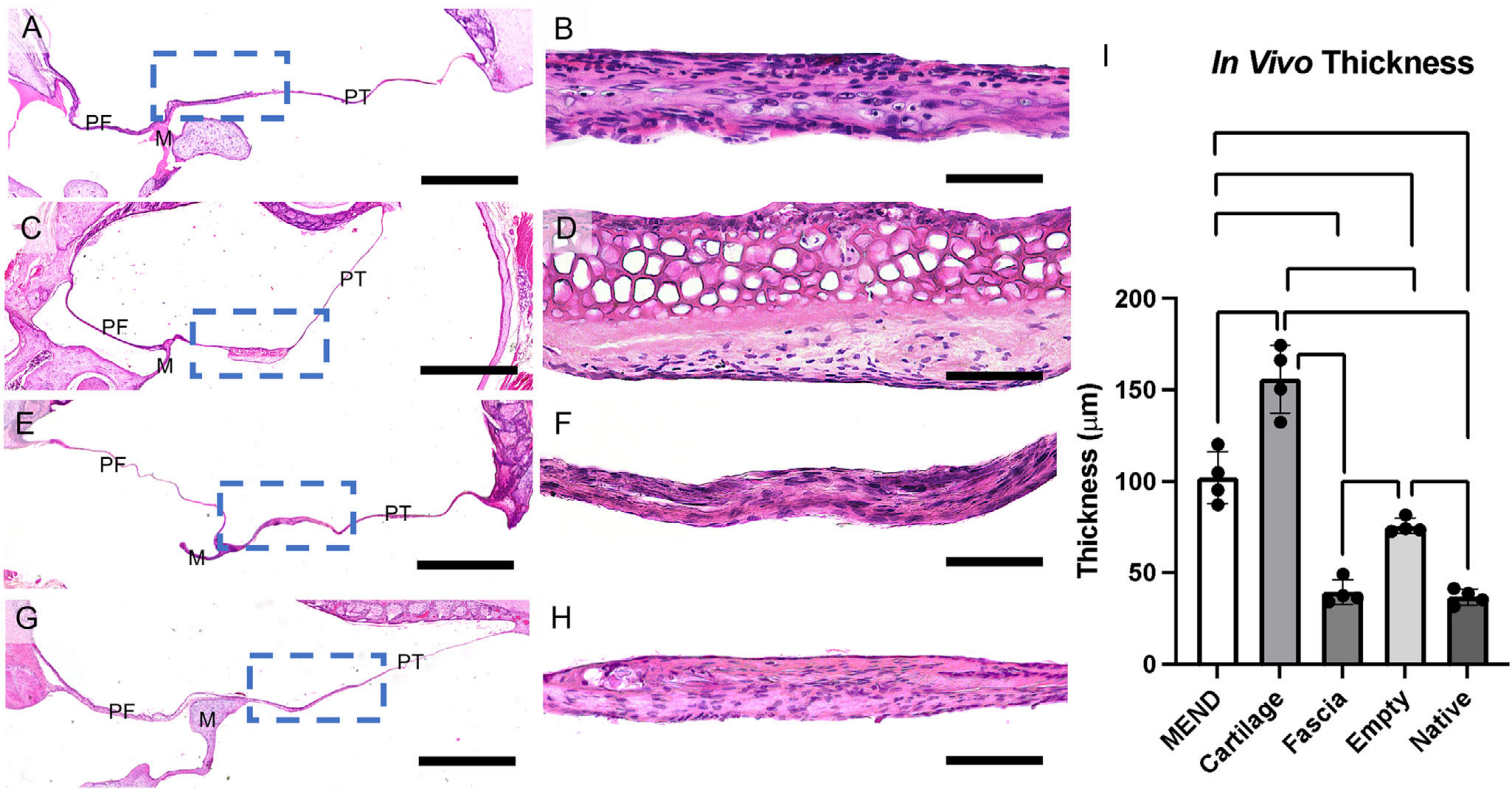
H&E stained tympanic membranes after 4 weeks with of in vivo implantation in a rat model of tympanic membrane perforation. (A,B) MEND demonstrates graft integration and significant remodeling. (C, D) Ear cartilage grafts show minimal host cell invasion and retention of native cartilage architecture. (E, F) Fascia grafts are readily accepted into the host tissue but present as a fibrous monolayer. (G, H) Rats receiving no graft (empty defect) healed at 4 weeks. Dotted box on the left column indicates zoomed in region on the right. (I) In vivo thickness of each graft after 4 weeks in vivo. PF = pars flaccida, PT = pars tensa, M = malleus. Right column: scale bars = 1 mm. Left column: scale bars = 100 µm, * = *p* < 0.05, ** = *p* < 0.01, **** = *p* < 0.0001. Statistical analysis: ANOVA with Tukey's *post hoc*.

At 4 weeks post‐operatively, each rat was euthanized, and their TMs were sectioned and stained. H&E for all conditions showed a thickened TM at the perforation site (Figure 5A–H). Rats receiving MEND grafts exhibited complete graft integration into the TM with significant remodeling. TM immunostaining for CD68, a pan‐macrophage marker, in rats receiving MEND grafts shows no significant difference in macrophage density compared to rats receiving no treatment, further demonstrating MEND graft acceptance (Figure S2). MEND was extensively infiltrated by host cells throughout the entire graft over 4 weeks in vivo (Figure 5A,B) and each layer of TM was restored. Immunostaining for pan‐cytokeratin in rats receiving MEND grafts shows strong tissue epithelization, suggesting graft acceptance and restoration of the layered TM structure (Figure S2). In contrast, the auricular cartilage graft displayed no signs of remodeling, with the original cartilage architecture still clearly recognizable, and was overall significantly thicker than the surrounding TM tissue (Figure 5C,D). The graft histology before and after implantation remained substantially unchanged, with little‐to‐no cell infiltration into the dense cartilaginous matrix. Fascia grafts restored the fibrous layer of the TM and appear as a monolayered structure; with unclear evidence of re‐epithelialization in histology (Figure 5E,F). These observations could be due to the delay in perforation closure compared to MEND. In rats receiving no treatment, each layer of the native TM structure was present in the repaired tissue (Figure 5G,H).

The thickness of each graft after 4 weeks in vivo was measured from 10 different sections in each rat to calculate overall TM thickness after healing at the site of graft implantation. Fascia and the empty defect resulted in the thinnest healed TMs. MEND, although with a greater thickness, was within the same order of magnitude as the empty defect with an average thickness of 92 µm. Auricular cartilage was the thickest, with an average of 161 µm (Figure 5I).

**FIGURE 6:**
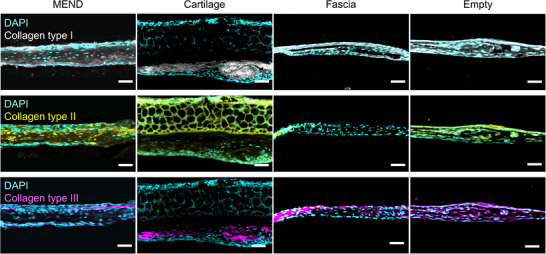
Immunofluorescence staining of collagen types I, II, and III of the MEND, cartilage, fascia grafts, and empty TM perforations at 4 weeks post‐implantation shows significant MEND and fascia graft remodeling, however, cartilage grafts did not remodel but rather retained their original architecture and collagen composition. Cyan = DAPI nuclei staining, Gray/White = collagen I, Yellow = collagen II, Magenta = collagen III. All scale bars = 50 µm.

Immunofluorescence was conducted to measure the matrix content within each graft after 4 weeks (Figure 6). After 4 weeks in vivo, MEND was fully remodeled with cells dispersed throughout the graft. Sparse amounts of collagen type I bundles from the native MEND graft were observed, suggesting that additional time may be required to allow for full graft remodeling over time. Collagen type II was abundantly present in MEND after 4 weeks in vivo but collagen type III was not at the same level as native TM at 4 weeks. Auricular cartilage grafts initiated a buildup of collagen I and collagen III below the graft, which may be indicative of fibrous wound healing. There is very low levels of cellular infiltration into the dense auricular cartilage graft, suggesting limited graft integration. As previously established, auricular cartilage is rich in collagen type II with an absence of collagen type I and collagen type III. Fascia grafts resulted in a healed TM containing mostly collagen type I and collagen type III. There is minimal collagen type II present within the graft area. Finally, the healed TM without a graft was positive for all three major collagen types, suggesting a restoration close to the native TM structure and composition.

## 4 Discussion

This study successfully demonstrates that MEND, a novel decellularized and channel‐laden fibrocartilage (MEND) scaffold, is a suitable graft material for pediatric TM repair. The clinical standards of care for pediatric TM repair are cartilage and fascia [11, 15, 16]. However, the challenging environment of the pediatric middle ear leads to high revision rates for both graft options [17]. Our study demonstrates that MEND may possess some of the advantages of both fascia and cartilage.

Our innovative process of selectively digesting the elastin fibers from the outer meniscus yields cartilage with homogeneously distributed channels, which allow for cellular invasion, graft integration, and remodeling (Figure 1A,B, I). Importantly, the selective digestion process maintained overall matrix structure and composition, resulting in retention of much of the mechanical stiffness of the original cartilage. During this process, we also successfully reduced the total DNA content below 50 ng/mg of tissue (Figure 1C). This previously established benchmark is essential for translational decellularized therapies, and notably, we achieved it without the use of harsh chemicals such as detergents [31, 33]. This likely contributed to the conserved mechanical stability of MEND post‐decellularization compared to auricular cartilage (Figure 2K) [34]. While several other groups have successfully engineered decellularized cartilage grafts by leveraging freeze‐thaw cycles and enzymatic digestion methods, these scaffolds are often subject to structural loss due to the decellularization process. In this work, we selected the meniscus as an ECM template rather than hyaline cartilage with the exact intent of leveraging selective elastin digestion to create a channel laden cartilaginous structure, to overcome some of the challenges associated with hyaline cartilage decellularization with a novel and innovative biomaterial strategy.

We used a rat model of TM repair to validate the viability of our engineered cartilage graft. Advantages of this model include the high accessibility of rat TM as well as the anatomical similarity between rat eardrum and the human. However, one drawback to the use of rats is the inability to create a critical size defect, as rat TM perforations reliably heal spontaneously (Figures 4 and 5G,H) [35]. In humans, by contrast, perforations requiring surgery are usually chronic and would not otherwise heal naturally. Nonetheless, this model is still widely used for studying middle ear disorders and can be very informative in testing graft effectiveness [36].

The great clinical need for eardrum repair procedures, coupled with the frequent need for revision procedures, has made TM graft engineering an emerging field, with researchers employing classic tissue engineering techniques and biomaterials to repair the TM. Materials such as hyaluronic acid, cellulose, collagen and chitosan have been used [20, 24, 37, 38]. However, difficulties with handling and poor mechanical strength have limited the clinical translation and overall efficacy of these materials for the pediatric population, as these grafts are unable to withstand the negative pressure often present in the pediatric middle ear. One study repaired a TM perforation with an injection‐molded calcium alginate hydrogel, and after 6 weeks, the TM healed around the hydrogel patch [20]. However, while promising for other tissue engineering applications, hydrogels remain far from clinical translation as pediatric TM graft options [39].

Patches or membranes, which mimic the fibrous nature of the TM, have also been proposed as potential grafts for TM repair. Since numerous collagen‐rich patches are already FDA approved, this approach is attractive from a clinical perspective. Decellularized patches derived from intestinal and urinary bladder submucosa are currently in clinical use as grafts for tympanoplasty [40, 41]. Decellularized urinary bladder matrix was used to repair TMs of chinchillas, with grafts reported to be adherent within one week and fully remodeled within the TM by 4 weeks. Histology showed complete integration as well as restoration of the trilaminar structure of the TM [40]. In the present work, we show that MEND achieved results similar to decellularized bladder matrix patches, albeit in the rat TM (Figures 5 and 6). Both technologies demonstrated improved integration and TM remodeling. Our data support the feasibility of decellularized scaffolds as an effective template for enhanced TM repair. However, MEND may be superior to alternative decellularized matrices derived from intestinal and urinary bladder submucosa since these have extremely limited mechanical properties, making them less than ideal for the challenging biomechanical environment of the pediatric middle ear [42]. Unlike those collagen‐based matrices, MEND grafts have better mechanical strength, analogous to that of cartilage, making them more amenable to pediatric TM repair given the need to withstand the negative pressure forces often found in children's ears. In addition, the more robust biomechanical properties of MEND allow it to be easily handled and manipulated by physicians during TM repair procedures, emphasizing the clinical translatability of this technology.

In our study, 28 rats were used to compare MEND to fascia, auricular cartilage, and a perforation healing without a graft. After 4 weeks post‐implantation, the attrition rate was 0%, and MEND grafts resulted in successfully closed perforations within 1 week. After 4 weeks, significant integration and remodeling were observed for MEND, in contrast to cartilage (Figures 3 and 5). While fascia grafts also integrated into the native TM, MEND closed perforations more rapidly than fascia and showed lower rates of surgical complications (Figures 3 and 4). MEND demonstrates the benefits of both cartilage and fascia without the known limitations of each of these grafts, suggesting that MEND is a promising alternative to the two most common solutions used for TM repair. In addition, MEND's thickness was substantially reduced from nearly 300 microns pre‐implantation (Figure 2J) to only 100 microns (Figure 6I), which more closely matches the thickness of the rat TM. These data provide evidence of successful MEND remodeling after in vivo implantation. Overall, MEND's ability to initially maintain its mechanical stability and avoid graft dislodgement, followed by its gradual remodeling into the native TM, represent distinct advantages over the current standards. Future studies will include use of larger animals, such as the chinchilla [43], for further validation of MEND in a model that allows evaluation in both acute and chronic TM perforations.

## 5 Conclusion

In this work, we applied a novel enzyme‐mediated method of cartilage decellularization to fabricate decellularized meniscal cartilage grafts rich in microchannels, which we termed MEND, for TM repair. MEND is a promising alternative to the current clinically available grafts for TM repair, because MEND not only is structurally similar to the native fibrous TM but also has the mechanical properties required to resist graft displacement. These qualities enable MEND to have wound closure rates faster than fascia and similar to cartilage, with high integration and remodeling that match the ideal properties of fascia. In fact, after 4 weeks post‐implantation in a preclinical rat model of TM perforation, MEND integrated into the native tissue as host cells invaded and remodeled the MEND grafts. Altogether, we demonstrate the potential for MEND as an off‐the‐shelf graft that shares the qualities of current clinical gold standards, fascia and cartilage, without the limitations of either, making it a promising solution to reduce surgical time and lower revision rates in pediatric tympanoplasty.

## Author Contributions

Conceptualization: PMG, JG, RG, Methodology: PMG, RMF, JG, Software: RMF, Investigation: PMG, RMF, GN, SF, DR, JG, Visualization: PMG, RMF, Writing – Original Draft: PMG, JG, RG, Writing – Review & Editing: PMG, RMF, JG, RG, Supervision: PMG, JG, RG, Funding Acquisition: PMG, RMF, JG, RG.

## Funding

Children's Hospital of Philadelphia Research Institute (RG, JG). Frontier Program in Airway Disorders of the Children's Hospital of Philadelphia (RG). National Science Foundation Graduate Research Fellowship Program No. DGE 1845298 (PMG, RMF). NIH NIAMS P30AR069619 for Core Facility use.

## Conflicts of Interest

Paul M. Gehret, Riccardo Gottardi are co‐inventors of a filed patent application for the technology used in this manuscript. All other authors declare that they have no competing interests.

## Supporting information




**Supporting File**: adhm71188‐sup‐0001‐FigureS1‐S2.pptx.

## Data Availability

All data are included in the manuscript.
